# A retrospective approach to assess human health risks associated with growing air pollution in urbanized area of Thar Desert, western Rajasthan, India

**DOI:** 10.1186/2052-336X-12-23

**Published:** 2014-01-09

**Authors:** Harcharan Singh Rumana, Ramesh Chandra Sharma, Vikas Beniwal, Anil Kumar Sharma

**Affiliations:** 1 Desert Medicine Research Centre (Indian Council of Medical Research), Jodhpur 342 005, Rajasthan, India; 2 Department of Biotechnology, Maharishi Markandeshwar University, Mullana-Ambala, Haryana 133203, India

**Keywords:** Air pollution, Human health, Environmental disease burden, Air quality

## Abstract

Air pollution has been a matter of great concern globally because of the associated health risks to individuals. The situation is getting worse in developing countries with more urbanization, industrialization and more importantly the rapidly growing population posing a threat to human life in the form of pulmonary, cardiovascular, carcinogenic or asthmatic diseases by accumulating toxic pollutants, harmful gases, metals, hydrocarbons etc.

**Objective:**

The present study was undertaken to assess the magnitude of ambient air pollutants and their human health risks like respiratory ailments, infectious diseases, cardiovascular diseases and cancer using a Retrospective Approach of Bart Ostra.

**Methodology:**

The parameters PM2.5, PM10, NOx, SO_2_, NH_3_ and O_3_ were monitored at all selected study sites monitored through a high volume sampler (APM 451 Envirotech, Envirotech Instruments Pvt. Ltd., New Delhi, India). Retrospective Approach was used for assessment of risk factors and disease burden of respiratory and cardiopulmonary health problems.

**Results:**

Environmental burden of disease showed that the problem of health related to air pollution is a main concern particularly in the growing cities of India. High to critical level of air pollution including PM10, PM2.5, NOx, SO_2_, NH_3_ and O_3_ was observed in all seasons at traffic intersections and commercial sites. The respiratory infections (25% incidence in population exposed to indoor smoke problems) and a prevalence of asthma/COPD (4.4%) in households exposed to high vehicular pollution along with signs of coronary artery/heart disease and/or hypertension and cancers (37.9-52.2%), were reported requiring preventive measures.

**Conclusion:**

The study reflects a great concern for the mankind with the need of having streamline ways to limit air pollution and emphasize upon efficiently determining the risk of illness upon exposure to air pollution.

## Introduction

The association of air pollution with human health is widely known as there are many health risks associated with the polluted environment. There has been continuous deterioration of ambient air and human health with the increase in population, industrialization and urbanization. Emission of toxic pollutants such as particulate matter (PM2.5, PM10 & TSP) and green house gases like ozone (O_3_), sulfur dioxide (SO_2_), Nitrous oxide (NO_2_) etc. have further aggravated this problem. Methane from swamps, livestock gases, cholorofluorocarbons (CFCs) used in refrigerants and aerosol propellants has further complicated the problem of deteriorating the earth’s ozone layer. There is a considerable risk to human health from these pollutants [1-7] and adequate information from the towns/cities of developing countries, where public health is a great concern, is required. All the particulate matters of varying sizes in combination with other air pollutants (gases, aromatic hydrocarbons, toxic metals etc.) have been found associated with health problems including aggravated asthma, lung cancer, chronic obstructive pulmonary disease (COPD), cardiovascular diseases and premature death [8].

The deteriorating air quality and air pollution has also been found to be associated with chronic morbidity, mortality and impacts on birth outcomes especially in traffic related pollution, releasing aerosols into the atmosphere [9-15]. These aerosols are made of mineral dust, sulfates, sea salt, or carbon etc. The aerosols comprise of dust lifted into the atmosphere from deserts, evaporating droplets from the ocean, the smoke from wildfires or erupting volcanoes. Burning of fossil fuels by humans also adds up to pollute the atmosphere. Computer models indicate that, worldwide, the tiny aerosols cause about half as much cooling as greenhouse gases cause warming. Air pollution can cause coughing, burning eyes, and breathing problems while in the long run may even contribute to life-threatening diseases such as cancer. The elderly, the young, and those with cardiopulmonary disease, or severe bronchitis, are the most vulnerable to air pollution exposure with children at a greater risk. In recent years interest has been focused on day-to-day particulate air pollution of fine particles-PM2.5 and coarse particles-PM10 in combination with toxic gases, leading to increased risk of adverse health outcomes. Each 10 μg/m^3^ elevation in fine particulate air pollution was associated with approximately 4%, 6%, and 8% increased risk of cardiopulmonary and lung cancer mortality respectively [16]. In addition, it has enhanced the risk for acute cerebro-vascular strokes [17,18], the aggravation of heart and lung disease and premature mortality [19-21]. The World Health Organization (WHO) estimated 84000 deaths directly attributable to outdoor air pollution in Indian cities.

The lack of services such as proper management of transport, paved roads and primitive roads and unplanned distribution of industries are all unable to keep pace with urban growth. All these in turn lead to an increase in the pollution levels. There is lack of such studies especially in India. The present study was undertaken to assess the magnitude of ambient air pollutants and their human health risks like respiratory ailments, infectious diseases, cardiovascular diseases and cancer using a Retrospective Approach of Bart Ostro.

## Materials and methods

### Study area and study sites

Jodhpur is a centrally located city in Rajasthan (India), where high industrial and commercial growth has been recorded since last decade. It is situated at latitude 26°18′N, longitude 73°04′E and an elevation of 230 m above msl (mean sea level) and expands over an area of 70 sq km and sub-divided into 60 wards by the Municipal Corporation. This is a valiant sentinel of the Thar Desert, which surrounds its northwest direction while the Aravalli hills are in its southeast direction. The wind blows usually from SW direction but during monsoon and winters, it blows from NE direction. The average horizontal light luminescence maximum in this city comprises of extreme dryness with intense hot summers, raising temperature to 49°C, accompanied by frequent dusty storms and modest temperature in winters with annual average rainfall of 31.0 cm. The study for existing air pollution monitoring was compared with primary data collected at Jodhpur with secondary data of other major cities like Ajmer, Kota, Jaipur including capital of India Delhi. Risk factors were assessed in all five major cities of India and their comparative account has been discussed. Different study sites were selected for assessment of commercial/vehicular, industrial and residential air pollution. Level of air pollution was separately studied for these categories because acceptable limits for each of them are different as given by Central Pollution Control Board (CPCB), Government of India.

### Ambient air quality monitoring

Different study sites were selected for assessment of commercial/vehicular, industrial and residential air pollution. The types of industries are related to dyes, textiles, timber and furniture, handicrafts, metals, chemicals, sandstones quarries, steel rolling mills, guar gum, pulses and oil mills etc. With Industries demand of transportation has also resulted massive addition of vehicles to the city where the residential areas too facing challenges throughout India.

Air quality monitoring at major 6 industrial sites, 9 traffic intersection sites and 14 residential sites on 8 hourly average bases were carried out during three seasons, covering winter, summer and rains (monsoon) to asses the magnitude of air pollution. The parameters PM2.5, PM10 (RSPM), NOx, SO_2_, NH_3_ and O_3_ were monitored at all selected study sites. A total of 45 samples from the industrial sites, 72 from the commercial/traffic intersection sites and 86 from the residential sites were collected during all the three seasons. These samples were analyzed using standard method as reported elsewhere [22-26]. The secondary data was also collected and pooled on similar pattern through correction factor from the secondary sources for other cities where RSPM and SPM was measured. RSPM was well defined as PM10 whereas SPM was total suspended particulate matter. Therefore, after applying correction factor derived from primary studies and secondary data, PM2.5 was assessed. The control group in such studies is not possible however, the background lowest concentration for criteria air pollutants as defined by EPA and adopted by CPCB are taken as threshold limit and therefore, the occurrence of disease prevalence.

### Sample collection and analysis

PM10 was monitored through a high volume sampler (APM 451 Envirotech, Envirotech Instruments Pvt. Ltd., New Delhi, India) in the ambient air. The instrument comprises of a protective housing, a high-speed electric motor blower, a filter holder for holding a 203 × 254-mm or 8″ × 10″ glass microfibre filters GF/A Whatman (Whatman International Ltd., England) a flow-controller which controls the flow rate at 0.9-1.4 m^3^/min over a minimal sampling period of 8 hrs as considered normal exposure work duration for inhabitants. The sampler was kept in the breathing zone at a height between 1.5-3.0 meters from the ground level at a central place. PM2.5 was measured through Envirotech air sampler APM 550. The APM 550 Envirotech air sampler is a microprocessor-controlled low-volume sampler (16.7 L/min), which measures logs flow rates and meteorological parameters. Ambient air passes through a PM2.5 sampling head where particles are being collected on a 2 μm PTFE 46.2 mm diameter (0.4 mm pore size) Whatman filter paper (Whatman International Ltd., USA). After collection of air samples, gravimetric method was applied for their analysis. The mass concentration of the suspended particulates was calculated and expressed in microgram per cubic meter air (μg/m^3^). Gaseous samples for SO_2_, NO_x_, NH_3_ and O_3_ were collected by impinger technique using a low volume sampler (Model EI Envirotech APM 411TE). For the collection of these samples, this sampling kit was attached with a high volume respirable dust sampler (EI Envirotech APM 451). The high volume sampler was kept for 8 hrs to sample the gaseous pollutants from various selected sites. Flow rate in collection of trace gases was maintained uniform, by frequently checking and correction of calibrated Rotameter. Impingers filled with reagents were placed in metal block kept in a cold box assembly. Temperature of block was reduced thermo-electronically and maintained constant inside the sampler at 15 ± 3°C using a thermostat. The samples collected were brought to laboratory and stored in the refrigerator at 4°C, before their chemical analysis. Sulfur dioxide (SO_2_) concentration was measured by the spectrophotometric method using TCM and fuchsin (West-Gaeke method). Nitrogen dioxide (NO_2_ -NO_x_) was measured by spectrophotometric method (Jakobs – Hochheiser method) using NEDA and sulfanilamide. Ammonia (NH_3_) and Ozone (O_3_) were measured by spectrophotometrically.

### Health assessment

Jodhpur is the largest district of Rajasthan comes under arid zone-Thar desert, whereas the urbanized area is second largest in the state afterwards of Jaipur. The urbanized area has population of about 1.0 million. Therefore, a population based cross sectional health survey was carried out in the entire city and its suburb covering all 60 wards (blocks) of the city i.e. upto municipal boundary limits. A sample size of approx 10,000 individual was taken to find out prevalence of morbidity conditions with prevalence of 1% or more, keeping standard error within 10%. Assuming at least six members in a household, it was decided to survey 1667 households from the city. Data related to environment related health problems at household level were collected in a pre-tested schedule provided by Ministry of Environment and Forests as well as from authenticated secondary records. The nomenclature of diseases and their definition is followed as described by the WHO which is common practice in Indian hospitals and dispensaries to maintain the records. The detailed information about number of patients treated at OPD or indoors regarding cancer, hypertension, respiratory diseases and cardiovascular diseases were also collected from Seven number Dispensaries, from office of Chief Medical and Health Officer of Jodhpur and from Principal of four teaching hospitals of Dr. S. N. Medical College, Jodhpur. Detailed analysis of data about health and diseases and their association with different environmental parameters has been carried out using software Epi Info 2000.

### Risk factor and environmental disease burden

A retrospective approach was used for assessment of risk factors and disease burden of respiratory and cardiopulmonary health problems. Recommended health outcomes and risk functions used to calculate the EBD has been shown in Table 1.

**Table 1:** Recommended health outcomes and risk functions used to calculate EBD

**Outcomes and exposure metrics**	**Relative risk function**^**a**^	**Suggested β coefficient (95% CI)**	**Sub-group**
All-cause mortality and short-term exposure to PM10^b^	RR = exp[β(X–X_0_)]	0.0008 (0.0006, 0.0010)^c^	All ages
Respiratory mortality and short-term exposure to PM10 (all-cause mortality for upper bound where applicable)	RR = exp[β(X–X_0_)]	0.00166 (0.00034, 0.0030)	Age <5 yrs
Cardiopulmonary mortality and long-term exposure to PM2.5 (log - linear exposure)^*^	RR = [(X + 1)/ (X_0_ + 1)]^β^	0.15515 (0.0562, 0.2541)	Age >30 yrs
Cardiopulmonary mortality and long-term exposure to PM2.5 (linear exposure)	RR = exp[β(X–X_0_)]	0.00893 (0.00322, 0.01464)	Age >30 yrs
Lung cancer mortality and long-term exposure to PM2.5 (log- linear exposure)^*^	RR = [(X + 1)/(X_0_ + 1)]^β^	0.23218 (0.08563, 0.37873)	Age >30 yrs
Lung cancer mortality and long-term exposure to PM2.5 (linear exposure)	RR = exp[β(X–X_0_)]	0.01267 (0.00432, 0.02102)	Age >30 yrs

^a^X = Current pollutant concentration (μg/m^3^) and Xo = target or threshold concentration of pollutant (μg/m^3^).^b^Not used in DALY calculations and should not be added to the other mortality estimates. ^c^Presentation of a range rather than a point estimate is preferred. ^*^Recommended relationships assuming background concentration for PM10 = 10 μg/m^3^ and for PM2.5 = 3 μg/m^3^.

### Calculating the disease burden

The relative risk for each included health outcome was calculated using the risk functions. Once the relative risks were determined, the AF –attributable factor (or IF impact factor) of health effects from air pollution for the exposed population was calculated by: AF = (RR – 1) / (RR). Secondary data was also collected from Seven Government Dispensaries out of total 23 dispensaries in the city. Secondary data regarding respiratory diseases, gastrointestinal diseases and cancers were also collected from all major hospitals in the city except the private hospitals and clinics due to lack of practice to maintain records.

## Results and discussion

The annual range, mean and standard deviation of respirable particulate matter up to size 10 microns (PM10), fine particulate matter up to size 2.5 microns deep penetration in lungs (PM2.5) and gases NO_x_, SO_2_, NH_3_ and ground level O_3_ of all selected sites for the industrial air pollution are given in Table 2. The exceedance factor (EF) for different sites to assess the pollution level was also calculated separately for each selected parameters. Forty-five samples were collected from the industrial sites for assessment of industrial air pollution in the city. The annual mean values of PM2.5 and PM10 were found within permissible limits of annual standards. The results shown in the Table 2 indicate that the concentration of both parameters was higher than the acceptable standards once or twice in a year indicating their seasonal effect and that was reported high during winters only. The higher level of air pollution during winter may be because of the high fuel usage in winter [27]. Trends of seasonal variation of all selected parameters were also observed. The levels of four gases at all industrial sites show that their EF values were below 0.5, indicating low level of pollution (Table 2). The data of NO_2_ and SO_2_ show declining trend from winter to rains whereas the values of ammonia and ozone were low in summers than in rains and winters. At all sites annual average of gaseous pollutants were found within the national ambient air quality standards (Table 2). The pooled and standardized average values of RSPM and SPM with their ratio (N = 185) in different cities of India have been shown in Table 3. Also the Environmental disease burden and Risk Functions of particulate matters in five cities of India has been illustrated in Table 4.

**Table 2:** Air pollution of different sources in the entire Jodhpur city

**Study areas**		**PM2.5**	**PM10**	**NO**_**x**_	**SO**_**2**_	**NH**_**3**_	**O**_**3**_
**Industrial sites**	Annual mean	71.4 ± 77.8	323.4 ± 236.2	23.44 ± 20.3	6.3 ± 5.64	26.4 ± 25.6	32.7 ± 25.1
	EF	0.6	0.9	0.3	0.1	0.3	0.2
	*Annual standard	120	360	80	80	100	120
**Traffic intersections/commercial sites**	Annual mean	143.8 ± 90.0	470.6 ± 234.5	52.0 ± 35.5	16.4 ± 11.5	61.2 ± 39.9	82.9 ± 56.2
	EF	2.4	3.4	0.9	0.3	0.6	0.5
	*Annual standard	60	140	60	60	100	157
**Residential sites**	Annual mean	54.3 ± 28.3	291.4 ± 129.6	15.4 ± 6.0	5.5 ± 2.3	35.6 ± 18.9	24.7 ± 11.9
	EF	0.9	2.1	0.2	0.1	0.4	0.2
	*Annual standard	60	140	60	60	100	157

*National Ambient Air Quality Standards-where standards considered for PM2.5--RSPM, for PM10-SPM and Ozone for 8 hours standards. Values of all parameters are in μg/m^3^ except the Exceedance Factor (EF).

**Table 3:** The pooled and standardised average values of PM2.5 and PM10 with their ratio (N = 185) in different cities of India

	**PM2.5**	**SD**	**PM10**	**SD**	**PM2.5:PM10**
Primary data					
Jodhpur	85.6	75.88	353.4	208.62	0.24
Secondary data					
Jaipur	94	64.0	227	118.1	0.41
Kota	96	55.9	253	143.0	0.38
Udaipur	94	56.6	277	135.9	0.34
NCR Delhi	171	108.0	433	173.0	0.39

*Primary data monitored for PM2.5 and PM10, **Secondary data for PM2.5 and PM10 pooled -standardized from RSPM and SPM.

**Table 4:** The Environmental disease burden and risk functions of particulate matters in five cities of India

Outcomes and exposure metrics*	**Sub-group**	**Risk function of particulate matter in some Indian cities (CI of 95%) and disease burden (AF)**									
		**Jodhpur**		**Jaipur**		**Kota**		**Udaipur**		**Delhi**	
		**RR (CI)**	**AF (IF)**	**RR (CI)**	**AF (IF)**	**RR (CI)**	**AF (IF)**	**RR (CI)**	**AF (IF)**	**RR (CI)**	**AF (IF)**
All-cause mortality and short-term exposure to PM10 (linear exposure)	All ages	1.29 (1.21-1.37)	0.22	1.19 (1.14-1.24)	0.16	1.22 (1.16-1.28)	0.18	1.24 (1.17-1.01)	0.19	1.40 (1.29-1.53)	0.29
Respiratory mortality and short-term exposure to PM10 (all-cause mortality for upper bound where applicable) (linear exposure)	Age <5 years	1.69 (1.11-2.57)	0.41	1.43 (1.08-1.92)	0.30	1.50 (1.09-2.07)	0.33	1.56 (1.10-2.23)	0.36	2.02 (1.15-1.27)	0.50
Cardiopulmonary mortality and long-term exposure to PM2.5 or (log- linear exposure)	Age >30 years	1.67 (1.20-2.32)	0.40	1.64 (1.20-2.24)	0.39	1.64 (1.20-2.25)	0.39	1.63 (1.20-2.24)	0.39	1.79 (1.24-2.60)	0.44
Cardiopulmonary mortality and long-term exposure to PM2.5 (linear exposure)	Age >30 years	2.56 (1.40-4.65)	0.61	2.25 (1.34-3.79)	0.56	2.30 (1.35-3.90)	0.56	2.25 (1.34-3.79)	0.56	4.48 (1.72-11.70)	0.78
Lung cancer and long-term exposure to PM2.5 or (log linear exposure)	Age >30 years	2.15 (1.33-3.50)	0.54	2.09 (1.31-3.32)	0.52	2.10 (1.31-3.35)	0.52	2.09 (1.31-3.32)	0.52	2.39 (1.38-4.16)	0.58
Lung cancer and long-term exposure to PM2.5 (linear exposure)	Age >30 years	3.78 (1.57-9.09)	0.74	3.17 (1.48-6.77)	0.68	3.25 (1.50-7.06)	0.69	3.17 (1.48-6.77)	0.68	8.40 (2.07-34.17)	0.88

*Recommended relationships assuming background concentration for PM10= 10 μg/m^3^ and for PM2.5= 3 μg/m^3^ in current perspectives.

Critical levels of air pollution were observed during peak traffic or rush hours particularly in winters at traffic intersections/commercial sites. The highest levels of PM2.5 and PM10 were found at the inner city roadsides. The levels of annual average of PM2.5 and PM10 in the breathing zones were also found to be high to critical level of pollution (Table 2). These traffic intersections were surrounded by residential settlements and population living there is likely to be exposed to these critical levels of air pollution. PM2.5 and PM10 pollution at traffic intersections/commercial sites were found high during all three seasons and varied between the categories of high to critical level. Their higher values along with higher to critical levels of PM2.5 and PM10 may have been caused by increasing number of vehicles in the city.

To assess the vehicular air pollution in the city, out of 56 samples collected from the commercial/traffic intersection sites (with nearby residential areas), 10 samples were collected in winters, 25 in summers and 21 during the rainy season on 8 hourly basis. It was observed that the annual averages of PM2.5 and PM10 at different sites were above the national ambient air quality standards. The annual average of PM2.5 and PM10 at all sites were reported 2–3 times higher than the annual standards as given in the Tables 2, 3, 4. PM2.5 and PM10 were higher in winters than summer and rain seasons, showing critical levels of air pollution. The seasonal trend of air quality (PM2.5, PM10, TSP) at traffic intersections and road corridors across the city indicate that it was higher throughout the year. The annual average of these four gaseous pollutants at vehicular/commercial sites of the city were found within the limits of annual standards except during winter season when these were higher (Tables 2, 3, 4). The seasonal observations of these gaseous pollutants showed a declining trend from winters to rains. The concentration of NOx, NH_3_ and O_3_ gases were observed higher than the national ambient air quality standards in winters but were with in the limits in summers and rainy seasons. This indicates the presence of winter smog at all the traffic intersection/commercial sites. A declining trend of O_3_, NO_x_ and SO_2_ from winter to summer and then to rains while level of NH_3_ was least in summers. Simultaneously, 84 air samples were collected from the residential sites during summer, rainy and winter seasons. The PM2.5 at all sites were found within limits of the national ambient air quality standards. The concentrations of PM10 in all study sites were found above the annual standards. Majority (71.3%) of households were exposed to medium level of vehicular traffic pollution, followed by 17.6% to low level and 11.1 percent of high vehicular pollution. Prevalence of asthma/COPD was 4.4% in households exposed to high vehicular pollution, 3.2% in those exposed to medium vehicular pollution and 2.6% in those exposed to low vehicular pollution (Table 5). Similarly 25.5% of the population felt indoor smoke problems where the prevalence of respiratory diseases was 8.1%, as compared to 6.2% (p = 0.001) in those not having indoor smoke pollution. Prevalence of tuberculosis was 1.5% in former group of households while it was 0.5% in later group (Chi square = 25.4; p = 0.00004) (Table 6).

**Table 5:** Prevalence of Asthma/COPD according to level of vehicular traffic pollution in houses

**Level of vehicular traffic pollution in House**	**Proportion of HH**	**Prevalence of asthma/COPD**
High	11.1%	4.4%
Medium	71.3%	3.2%
Low	17.6%	2.6%

**Table 6:** Prevalence of respiratory diseases according to level of indoor air pollution

**Level of indoor air pollution in-house**	**Proportion of HH**	**Prevalence of Resp. Dis.**	**Prevalence of Pulm. TB**
High	25.5%	8.1%	1.5%
Low	%	6.2%	0.5%

It is evident that southwest winds might also contribute to higher levels of pollution in the city. According to our surveillance report about 8.6% of the population had the industrial air pollution in their surroundings (3.6% mines, and 5.6% other industries). Secondly annual standards for industrial areas are more than double of commercial or residential sites. The values of PM2.5 and PM10 remain high here throughout the year in terms of the population exposure. In the 8.6% population, which had the industrial air pollution in their surroundings, prevalence of respiratory diseases was 9.9% as compared to 6.4% in remaining population (p = 0.005). The size of the particle is quite significant because particles < 2–3 μm in aerodynamic diameter tend to deposit deep in the lungs, in terminal bronchioles and alveoli while large particles tend to deposit in the upper airways [28]. TSP includes all suspended particles up to 30–40 μm size [29]. This inhalable fraction is equivalent to delivered dose for particles greater than approximately 25 micron (aerodynamic particle diameter), which get deposited completely and almost exclusively in the extrathoracic airways.

Prevalence of respiratory and cardiovascular diseases including hypertension may be attributed to the higher levels of air pollution in the city. Based on their housing details, 2.9% of individuals were slum dwellers (lowest income group), 46.7% lived in low income settlement, 47.3% in middle-income settlement and 3% lived in posh colonies (high income settlement). These settlements could vary in indoor air pollution, as 25.5% of the population felt indoor smoke problems. About 73.1% population solely used LPG as fuel in the households, where as about 14.1% used only biomass (wood, dried cow dung etc.) and about 3% depended on combination of kerosene/biomass/LPG. The association between fuel used and prevalence of respiratory diseases are mentioned in Table 7. Maximum prevalence of respiratory diseases (10%) was in the population, using biomass as fuel. Prevalence of respiratory diseases was comparatively lower (6.1%) among LPG users. Personal habits including smoking could also have contributed to causation of respiratory diseases. Prevalence of smoking was 3.1% (3.0% male, 0.1% female). Of all 3.0% (2.1% males, 0.9% females) used oral tobacco (zarda) and 5.9% (4.3% males, 1.6% females) consumed gutka; while 88.0% of population was not addicted to such habits. The population density was also very high around the traffic intersections/commercial sites. The narrow width of roads and tall buildings further aggravated the pollution levels.

**Table 7:** Prevalence of respiratory diseases according to fuel used

**FUEL**	**Prevalence of respiratory diseases (Percent)**	**Fuel users**	
		**No.**	**Percent**
Biomass	10.0	1306	14.1
Kerosene	7.9	76	0.8
Kerosene/biomass	8.6	243	2.6
LPG	6.1	6792	73.1
LPG/biomass	5.6	715	7.7
LPG/kerosene	8.1	86	0.9
LPG/kerosene/biomass	4.3	69	0.7
TOTAL	6.7	9287	100.0

The gaseous air pollution at residential sites was also measured and it was found within range of the national ambient air quality standards. The EF was also calculated and all values were below 0.5 indicating low level of air pollution. Annual averages of all gases at residential sites were found within the limits of national ambient air quality standards. The EF values of NOx, SO_2_, NH_3_ and O_3_ were 0.2, 0.1, 0.4 and 0.2 observed respectively, indicating low level of gaseous air pollution. Higher concentration of PM10 was found at residential sites (Table 2). PM2.5 was ranging between low levels to moderate level of air pollution. The pollution was found high at those residential sites, which are influenced by movements of pollutants generated from free flowing traffic road corridors or influenced nearby the stone quarrying mines or by other activities. The levels of PM2.5 and PM10 at industrial sites were higher in winters (EF = 1.3) (Tables 2 and 3) though their annual means were within the limits of annual standards. Similar observations about PM2.5 and PM10 have been made earlier as well [30-32]. Kumar and Joseph [33] were also reported that the concentration of PM2.5, PM10, and NO_2_ in a residential cum commercial area of Mumbai city in India. PM2.5 levels also exceeded 24 hourly average USEPA standard (U.S. EPA, 2004) during winter season indicating unhealthy air quality. Present study revealed moderate to critical levels of PM10 and PM2.5 in Jodhpur and CPCB has also depicted similar levels of PM10 and PM2.5 in five cities of Rajasthan, India (http://www.cpcbedb.nic.in). Arena et al. [34] reported that there is a positive association of PM10 with hospital admissions, and the effect is related to current-day PM10 levels. The air pollution-cardiac disease association was not significantly influenced by gender or community level of education or income [35]. The earlier reports also established an association between air pollutant levels and abnormal ventilatory functions in three sites of Mumbai city in India near industrial, commercial and residential areas [36]. A high prevalence of approximately 30-50% of respiratory symptoms was reported. However, In Jodhpur city, respiratory diseases were higher in the commercial areas, which are associated with the higher mean and peak levels of SO_2_ and the NO_2_. The pollution control measures should also aim at the peak levels of pollutants as they have been shown to exacerbate the respiratory symptom [36]. In the present study prevalence of asthma/COPD (Chronic Obstructive Pulmonary Diseases) was 3.4%, which is close to prevalence of chronic bronchitis (2.3-5.0%) in four areas of Mumbai city [37] and 3.3% in North Ambedkar district in Tamil Nadu [38]. In a multi-centric study in India, prevalence of COPD (chronic obstructive pulmonary disease) was 4.1%, with a male to female ratio of 1.56:1 and a smoker to non-smoker ratio of 2.65:1 in urban and the rural populations at Bangalore, Chandigarh, Delhi and Kanpur [39], which is quite in agreement with our observations. Overall the approach used in the study has been quite successful, despite few limitations such as 1) the actual magnitude of the effect and the appropriate confidence interval show little uncertainty 2) Also some of the estimated health effects may include other co-pollutants as well affecting the outcome 3) Additionally one has to assume a baseline incidence level for the city and the incidence change over time as health habits, income and other factors change.

## Conclusions

High to critical level of air pollution including PM10, PM2.5, NOx, SO_2_, NH_3_ and O_3_ was observed in all seasons at traffic intersections and commercial sites, more so in winter with majority of households exposed to medium level of vehicular traffic pollution. There appeared to be a prevalence of asthma/COPD (4.4%) in households exposed to high vehicular pollution. Similarly 25.5% of the population which was exposed to indoor smoke problems, there was a major incidence of respiratory diseases. Some of the population also showed a history of coronary artery/heart disease and/or hypertension, COPD/asthma, acute lower respiratory infections and cancers which might in part be due to built up of gaseous and particulate pollutants. Environmental burden of disease showed that the problem of health related to air pollution is a main concern particularly in the growing cities of India. About 37.9-52.2% of the population under study was found to be facing the cardiopulmonary and lung cancer risks (age group >30 years) which require immediate preventive measures. The study will further guide us to assess the health risks associated with polluted environments especially in children to find the relative respiratory and other risk functions in them.

## Abbreviations

PM: Particulate matter; PM10: Particulate matter of equivalent or upto 10 micron size; PM2.5: Particulate matter of equivalent or upto 2.5 micron size; AFs: Attributable fractions; EBD: Environmental burden of the disease; CFCs: Chlorofluorocarbons; CHD: Coronary heart disease; TSP: Total suspended particles; COPD: Chronic obstructive pulmonary disease.

## Competing interests

Authors declare that there exist no potential conflict of interest or competing interests regarding publication of the said manuscript.

## Author contributions

Following are the details of the contributions made by each of the authors for the manuscript: Dr. HSR, PhD was the key person who worked diligently to furnish this study and manuscript. He performed each of the experiments with his own hands. Dr. RCS, D.Sc was the principal investigator of the Project and majority of the experiments were done under his supervision. Dr. VB analyzed part of the findings referred in this study and helped in preparation of the manuscript. Dr. AKS was involved in analysis part of the data pertaining to this study and was instrumental in preparing and reviewing this manuscript including tables and figures. All authors read and approved the final manuscript.
